# Indigenous Knowledge of Farmers in Breeding Practice and Selection Criteria of Dairy Cows at Chora and Gechi Districts of Ethiopia: An Implication for Genetic Improvements

**DOI:** 10.1155/2022/3763724

**Published:** 2022-02-18

**Authors:** Gelaye Gebisa Bulcha, Oda Gizaw Dewo, Mengistu Asrat Desta, Chiemela Peters Nwogwugwu

**Affiliations:** ^1^School of Animal and Range Sciences, Hawassa University, Hawaasa, Ethiopia; ^2^Department of Animal Science, Mettu University, Mettu, Ethiopia; ^3^Department of Animal Science, University of Calabar, Calabar, Nigeria

## Abstract

This study assessed the breeding practice and selection criteria of dairy cows in two districts. A total number of 288 structured questionnaires were utilized to gather information from households in the study areas. Logit model, indices, and descriptive statistics were employed for data analysis. Education, marital status, and family size of respondents from Chora district were confirmed as predictors for practicing the controlled mating system and significantly influenced at *p* < 0.05. The odds of practicing the controlled mating system by educated and married farmers in Chora district were 10.01 and 4.82 times higher compared to uneducated and unmarried farmers, respectively, and also, for every additional increase in family size, they increased by the factor of 1.21. Educational and marital status of farmers in Gechi district also influenced the use of controlled mating. The odds of performing controlled mating based on the educational level and marital status of the farmers were higher among educated and married individuals. Based on indigenous knowledge, teat size, udder size, and pelvic width were the 1^st^ three ranked traits used as major selection criteria of dairy cows in Gechi district, whereas body length was the 1^st^ among others in Chora district. This finding indicated that the combination of indigenous knowledge with modern science is important to improve cow's genetics. The study suggests that mating systems and selection criteria should be considered as baseline information for designing the genetic improvement programs.

## 1. Introduction

Livestock farming is strongly associated with rural communities because of the importance of livestock in the society [1]. Livestock species have been selected based on the needs and agroclimatic conditions of the region. Some of these breeds of livestock have been developed based on their importance, adaptation, and availability in that region and also their ability to be improved by selection. Introduction of improved selection methods and exotic breeds around the world such as Asia, Africa, and Latin America has changed the traditional knowledge and skills as well as traditional selection practices of livestock farmers [1]. Indigenous technical knowledge (ITK) is a vital part of beliefs and antiquity of indigenous societies, and it has advanced over the years of consistent trailing on the routine life and existing assets in the community. It is also indispensable for the conservation of genetic resources for the sustainability of the breed/type [2].

Ethiopia is greatly rich in cattle population in Africa, and it is endowed with a wide range of local genetic resources and diverse livestock production systems [3]. The estimated cattle population in percentage in Ethiopia is 97.76 (indigenous), 1.91 (hybrid), and 0.32 (exotic) [4]. There are about 32 identified local breeds of cattle; however, the indigenous knowledge on livestock genetic resource management was unreported in Ethiopia [3]. Milk, meat, income, and other social functions are the primary aim for keeping cattle in Ethiopia. Some of these cattle are characterized with low productive performance. Low productive performance of cattle may be due to the influence of limiting factors such as lack of genetic improvement interventions, input shortage/lack, and indigenous farming practices as well as other environmental factors [5, 6]. Survivability and adaptability are the main preferred traits by the farmers. These traits enable the local breeds of cattle to survive and thrive on harsh environmental conditions.

In dairy cattle breeding, the indigenous breeding bulls have been used for natural mating. This method is practiced by most of the dairy farmers dwelling in highlands, midlands, and lowlands of Ethiopia [5], whereas artificial insemination (AI) has been used by some of the farmers in some regions. Farmers and their trait preferences differ across communities, farming systems, and agroecological zones [7]. Livestock production system influences the ranking system of a specific trait by livestock keepers. It has been reported that the livestock production system has a direct influence on some of the economically important traits. Furthermore, traditional knowledge and skills used to produce viable livestock rearing practices such as animal husbandry practices [8]. Studies on traditional cattle breeding practice and selection criteria are limited in some districts of Oromia Regional State of Ethiopia. This research therefore is aimed at investigating the indigenous breeding practice and selection criteria of cattle practiced by farmers in Chora and Gechi districts of Ethiopia.

## 2. Materials and Methods

### 2.1. Description of the Study Areas

The study was conducted at Gechi and Chora districts of Oromia Regional State of Ethiopia. Districts are characterized with good potential livestock production and some cash crop production. Gechi and Chora districts are situated at 462 and 516 km from the capital city of Addis Ababa towards the southwest direction of the country [9]. Gechi district is located at the longitude of 36° 39′ 59.99″ E and latitude of 8° 19′ 60″ N [10] and Chora district at 36°14′60″ E and 8°19′60″ N [11]. Furthermore, Chora district is popularly known as khat (*Catha edulis*) and coffee (*Coffea*) producing district besides livestock [5]. The authors further reported that agriculture is the main source of livelihood in both districts, and mixed livestock and crop production systems are well practiced in the study areas.

### 2.2. Sample Size Determination

The total number of respondents required was determined using the formula developed by Cochran [12] for the heterogeneous populations. The formula is

(1) $$\begin{array}{c} N=\frac{Z^{2}pq}{e^{2}}, \end{array}$$

where *N* is the total number of desired respondents, *Z* is the standard deviate value, *p* is the proportion of the study population from the entire population, *q* is the complementary proportion to *p*, and *e* is the desired absolute precision. Based on the values of *Z* = 1.96, *p* = 0.25, *q* = 0.75, and *e* = 0.05, a total of 288 households were involved in this study.

### 2.3. Sampling Technique and Data Collection

Multistage sampling techniques were employed to select the representatives from the study areas. Among the existing districts in Buno Bedele Zone, Gechi and Chora districts were selected based on livestock population potential and accessibility of infrastructures such as roads and others. The list of farmers involved in cattle rearing with a minimum of five years was compiled through consultation and assistance of agricultural experts in both study areas. Among a total of 288 households that participated in the study, 144 of them were selected for an interview section from each district using proportional random sampling techniques. The proportion was conducted based on the area covered in different agroecological zones. Primary data were collected using a structured questionnaire, whereas secondary data were from different governmental offices and other sources. The questionnaire was pretested before the real data collections. The interview was conducted on the general household's characteristics, cattle mating systems, and indigenous knowledge for dairy cow selection.

### 2.4. Data Management and Analysis

The data collected were prepared using a Microsoft Excel sheet and analyzed using R software package, version 4.0.3 [13]. Descriptive statistics (%) were employed to determine the proportion of mating systems. The association between general household characteristics and the controlled mating system was determined by multiple binomial logistic regression analysis. The selection criteria of dairy cows were calculated using indices employed by Musa et al. [14] for ranking different parameters. The calculation was performed to assess the ranking of household's responses on criteria of selecting dairy cows. The formula is

(2) $$\begin{array}{c} \text{Index}=\frac{\text{Sum of particular selection criteria}\left(R_{n}{}^{*}C_{1}+R_{n-1}{}^{*}C_{2}+\ldots \ldots \ldots +R_{1}{}^{*}C_{n}\right)}{\text{Sum of all selection criteria }\left(R_{n}{}^{*}C_{1}+R_{n-1}{}^{*}C_{2}+\ldots \ldots \ldots +R_{1}{}^{*}C_{n}\right)}, \end{array}$$

where *R*_*n*_ is the last rank (for example, if the last rank is 8, then *R*_*n*_ = 8, *R*_*n* − 1_ = 7, and *R*_1_ = 1), *C*_*n*_ is the frequency of respondents in the last rank, and *C*_1_ is the frequency of respondents ranked first. From the above formula, the “numerator” represented the sum given for particular selection criteria, and the “denominator” is the sum given for all selection criteria.

### 2.5. Model

The generalized multivariate binomial logit (G-MBL) model was employed to identify the association between factors influencing the mating system. The model was fit to dependent variables (controlled and uncontrolled mating systems) and also determined the effect of independent variables. The model was described as follows:

(3) $$\begin{array}{c} P\left(Y=1\right)=\frac{1}{1+e^{-\left(\beta_{0}+\beta_{1\times 1}+\beta_{2\times 2+\cdots \cdots +\beta_{p\times p}}\right)}}, \end{array}$$

where *P*(*Y* = 1) is the probability of being a controlled mating system, *X*_1 − *p*_ is the vector of predictor variables (sex, age, marital status, educational status, and family size), *β*_0_ is the intercept of the equation, and *β*_1_, *β*_2_,… *β*_*p*_ are coefficients of predictor variables.

## 3. Results and Discussion

### 3.1. Breeding Practice

**Figure 1:**
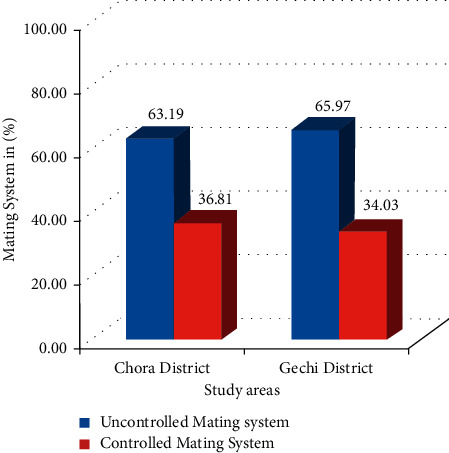
Proportion of controlled and uncontrolled cattle mating systems in the study areas.

The breeding practice of households in the study areas involves controlled and uncontrolled mating systems (Figure 1). The result indicates that the uncontrolled mating system was dominantly practiced by 63.19% of farmers in Chora and 65.97% in Gechi districts. Mating system plays an important role on the livestock improvement scheme, and the use of natural bull service through uncontrolled or unplanned mating is rampant in both study areas. Similar results have been reported by Ayantu et al. [15] in Western Oromia, Ethiopia. Our results agree with those of Azage et al. [6], who observed that free range mating is predominant in the rural areas of Ethiopia. This could be attributed to the lack of awareness, inaccessibility of information, and lack of adhering advice provided by agricultural extension workers. It could also be due to the loss of trust while using AI and absence of selected bulls for natural mating. Mengistu et al. [5] reported similar results in the Bedele district of Oromia Regional State of Ethiopia.

### 3.2. Factors Affecting the Mating System

The relationship between factors affecting the mating system in Chora and Gechi districts is presented in Tables 1 and 2. The result indicates that educational status, marital status, and family size are predictors for applying the controlled mating system in Chora district. The factors significantly influence those farmers who engaged in the controlled mating system. The findings further indicate that educational status of the farmers was highly significant among the factors. The odd of practicing controlled mating was 10.01 higher in educated farmers compared with uneducated farmers. This implies that the rate of transfer and adoption of new technology are higher among educated farmers. Similar trends were observed between married and unmarried as well as family size. It was evident that family size is associated with the use of controlled mating. In Gechi district, similar results and trends were observed but with different magnitudes in educational and marital status. The findings showed that educated and married farmers are highly engaged in the controlled mating system. These factors are indicators of the controlled mating system in both regions. However, an increase in family size did not encourage the controlled mating system in Gechi district. The impact of educational status of farmers on adoption of advanced technologies has been examined in previous studies [16, 17]. The finding indicates that educated households are knowledgeable in decision-making towards the controlled mating system in terms of cows' reproduction. This could be attributed to that educated farmers are faster to learn advanced technologies, accept, and implement advices from professionals. Oduro-Ofori et al. [18] stated that formal education is important for improving agricultural products and productivity through opening the minds of farmers to knowledge. The authors added that informal education keeps the farmers well informed with changing innovations and ideas and allows farmers to share their gained experience. The finding reported by Paltasingh and Goyari [19] agrees with our study and supports a previous study [17]. Our results revealed that married households are engaged with the controlled mating system, and it may be due to the fact that the married households have more concern for reducing the consequence of inbreeding and increasing the production to maintain their livelihoods. Similar results have been reported by Mabe et al. [20].

### 3.3. Selection Criteria of Dairy Cows

Several traits were observed as selection criteria in dairy cows among the farmers dwelling in Gechi district (Table 3). Our result revealed that teat size, udder size, pelvic width, body length, neck size, height at wither, navel flap size, and dewlap size were ranked from 1^st^ to 8^th^ with index values of 0.191, 0.182, 0.153, 0.13, 0.12, 0.116, 0.068, and 0.04, respectively.  Table 4 shows indigenous selection criteria of dairy cows in Chora district. The findings showed that body length, udder size, pelvic width, teat size, height at wither, navel flap size, neck size, and dewlap size were ranked from 1^st^ to 8^th^ with index values of 0.167, 0.166, 0.138, 0.132, 0.124, 0.117, 0.091, and 0.066, respectively. Based on the selection criteria, the first three traits are important and preferred by farmers. Those traits are indicators for selecting dairy cows. Several authors have reported long teat as an indicator for dairy cows [1, 21]. The authors added that cows with long teat are less vulnerable to mastitis. Long teat channel prevents infectious diseases because of the teat channel cessations after milking. It also stops the association between udder tissues and the external environment [22]. Udder size and pelvic width were also observed as selection criteria for dairy cows. Several authors have reported udder size and pelvic width as selection criteria for dairy cows in Northern Amhara Region, Northern Gondar Zone, and East Gojjam Zone of Ethiopia [23–25]. More recently, udder size has been reported by Soeharsono et al. [26], who observed a correlation between udder size and milk production. The correlation may be due to the fact that milk secretion is highly associated with the development of the mammary gland. Our findings are in agreement with the previous finding of Gorewit [27]. Wider pelvic size was also observed as a selection criterion, and this implies that farmers prefer cows with wider pelvic size to prevent difficulties in calving (dystocia). Similar results have been reported by Godadaw et al. [23], Ayeneshet et al. [24], and Andarge et al. [25]. Body length was observed as one of the selection criteria for dairy cows in Chora district. Yeman et al. [28] reported similar results in Gondar, Ethiopia. Moreover, Bayram et al. [29] observed that body length is associated with milk production.

## 4. Conclusion

In both study areas, the farmers were predominantly engaged with the uncontrolled mating system. The variables such as educational status, marital status, and family size were predictors of the controlled mating system in Chora district, whereas family size was not an indicator of controlled mating in Gechi district. This study showed that indigenous knowledge for dairy cow selection is commonly practiced in the study areas, suggesting that predictor variables, breeding practice, and selection criteria should be considered in designing dairy cows' genetic improvement scheme. This study suggests that awareness should be created on the effect of the uncontrolled mating system across productive and reproductive performances of cattle and its consequences.

## Figures and Tables

**Table 1:** Estimates and odds ratio of factors affecting controlled mating systems at Chora district.

Variables	G-MBL model			
	Estimate	Std. error	*p* value	Odd ratio (95% CI)
Sex (male)	0.2281	0.6401	0.722	1.26 (0.35 to 4.44)
Age	−0.0166	0.0302	0.582	0.98 (0.93 to 1.04)
Educational status (educated)	2.3032	0.6534	0.000^*∗∗∗*^	10.01 (3.16 to 44.59)
Marital status (married)	1.5732	0.7887	0.046^*∗*^	4.82 (1.12 to 26.39)
Family size	0.1940	0.0924	0.036^*∗*^	1.21 (1.02 to 1.47)

The *p* value <0.05 tells a significant difference. Significant codes: ^*∗∗∗*^ = 0.001, ^*∗∗*^ = 0.01, and *∗* = 0.05.

**Table 2:** Estimates and odds ratio of factors affecting controlled mating systems at Gechi district.

Variables	G-MBL model			
	Estimate	Std. error	*p* value	Odd ratio (95% CI)
Sex (male)	0.6051	0.568	0.287	1.83 (0.60 to 5.75)
Age	−0.0320	0.022	0.154	0.97 (0.92 to 1.01)
Educational status (educated)	1.6926	0.496	0.001^*∗∗∗*^	5.43 (2.16 to 15.42)
Marital status (married)	1.6817	0.688	0.014^*∗*^	5.37 (1.50 to 23.26)
Family size	0.1561	0.118	0.185	1.17 (0.93 to 1.48)

The *p* value <0.05 tells a significant difference. Significant codes: ^*∗∗∗*^ = 0.001, ^*∗∗*^ = 0.01, and *∗* = 0.05.

**Table 3:** Selection criteria of dairy cows in Gechi district.

Traits	Proportions								Index	Rank
Teat size	14	20	12	14	46	26	14	0	0.191	1
Udder size	2	28	28	56	8	10	12	0	0.182	2
Pelvic width	2	30	30	30	22	14	20	0	0.153	3
Body length	66	0	2	0	8	18	14	38	0.13	4
Neck size	12	12	20	0	24	44	28	2	0.12	5
Height at wither	14	2	22	0	30	30	32	10	0.116	6
Navel flap size	0	52	26	44	0	2	20	0	0.068	7
Dewlap size	34	0	4	0	6	0	4	94	0.04	8

Index = [(8 for rank 1) + (7 for rank 2) + (6 for rank 3) + (5 for rank 4) + (4 for rank 5) + (3 for rank 6) + (2 for rank 7) + (1 for rank 8)] for each of the traits divided by sum of all the traits.

**Table 4:** Selection criteria of dairy cows in Chora district.

Traits	Proportions								Index	Rank
Body length	18	0	6	14	44	12	22	22	0.167	1
Udder size	18	18	8	12	0	8	60	24	0.166	2
Pelvic width	20	30	4	44	8	40	12	0	0.138	3
Teat size	4	38	16	30	30	10	4	0	0.132	4
Height at wither	38	0	38	6	38	14	0	10	0.124	5
Navel flap size	18	38	42	2	0	24	38	0	0.117	6
Neck size	10	20	26	34	20	6	6	6	0.091	7
Dewlap size	18	0	4	2	0	30	2	82	0.066	8

Index = [(8 for rank 1) + (7 for rank 2) + (6 for rank 3) + (5 for rank 4) + (4 for rank 5) + (3 for rank 6) + (2 for rank 7) + (1 for rank 8)] for each of the traits divided by sum of all the traits.

## Data Availability

The datasets used for this study are available from the corresponding author.
